# Immunoevaluation of a Prokaryotic-Expressed Goose Circovirus Capsid Subunit Vaccine

**DOI:** 10.3390/microorganisms14061227

**Published:** 2026-05-29

**Authors:** Wenchang Xue, Chao Wang, Zhanxin Yao, Jialong Chen, Jipei Zhang, Jidang Chen

**Affiliations:** 1 School of Animal Science and Technology, Foshan University, Foshan 528225, China; 2112359034@stu.fosu.edu.cn (W.X.);; 2 College of Animal Science and Veterinary Medicine, Shandong Agricultural University, Taian 271018, China; 3 Agricultural Teaching and Research Bases, Foshan University, Foshan 528225, China

**Keywords:** *goose circovirus*, capsid protein, subunit vaccine, immunoevaluation

## Abstract

To address the lack of a commercially available vaccine for *goose circovirus* (GoCV), we developed and evaluated a prokaryotically expressed subunit vaccine targeting the viral capsid (Cap) protein. A truncated Cap protein (GoCV-ΔCap) was expressed in *Escherichia coli* (*E. coli*) and formulated with aluminum hydroxide as a subunit vaccine (GoCVsubvac). Goslings were primed intramuscularly (i.m.) with high (75 µg) or low (15 µg) doses GoCVsubvac, followed by a boost 14 days later. At 14 days post-boost, goslings were challenged with GoCV and were administered a bivalent inactivated vaccine against Newcastle disease virus (NDV) and H9-subtype Avian influenza virus (AIV). Using our established gosling pathogenicity model, vaccine efficacy was evaluated via body weight, lesions, viral load, antibody titers, cytokine responses, and interference with NDV/AIV immunity. Results demonstrated that the GoCV-ΔCap vaccine, especially the high-dose formulation, provided effective immunoprotection. It elicited robust humoral and cellular immune responses, reduced lymphoid pathology, and decreased the viral detection rate in lymphoid tissues from 100% (5/5) in infected controls to 40% (2/5). Importantly, it alleviated GoCV-induced immunosuppression and preserved the immunogenicity of co-administered vaccines. This novel subunit vaccine is a promising candidate for controlling GoCV disease (GoCVD).

## 1. Introduction

*Goose circovirus* (GoCV) is a non-enveloped virus with a 1.8 kb circular single-stranded DNA genome [1], ranking among the smallest known animal viruses. The GoCV genome contains four major open reading frames (ORFs): ORF V1, ORF C1, ORF C2, and ORF V2. Among these, ORF V1 encodes the replication-associated protein (Rep), comprising 293 amino acids and featuring a highly conserved amino acid sequence, which is essential for viral replication [2]. ORF C1 encodes the capsid protein (Cap), a 250-amino-acid structural protein that constitutes the viral nucleocapsid. Recognized as the major immunogenic component of GoCV, the Cap protein plays a pivotal role in eliciting virus-specific neutralizing antibodies (NAbs) and protective immunity [3,4].

GoCV was first identified in a goose flock in Germany in 1999 [5], subsequently reported in Hungary, the United Kingdom, Poland, and other countries [6,7,8]. In China, it was first detected in a goose flock in Taiwan Province in 2003 [9]. Although the direct mortality induced by GoCV is generally low, the virus inflicts substantial economic losses on the waterfowl industry through the induction of severe immunosuppression [10]. Infected geese are highly susceptible to secondary infections, and coinfections with pathogens such as *Avian influenza virus* (AIV) and *Newcastle disease virus* (NDV) are prevalent, further exacerbating disease severity and economic damage [11,12,13]. Given these implications, the development of an effective vaccine is critical for the sustainability of goose production.

The absence of a robust in vitro culture system for GoCV has impeded the development of conventional live-attenuated or inactivated vaccines. Subunit vaccines thus emerge as a viable and safe alternative, particularly those targeting the major structural capsid (Cap) protein, the primary antigen responsible for inducing neutralizing antibodies and capable of assembling into virus-like particles (VLPs) that elicit protective immunity [14,15]. For related *circoviruses* (e.g., *Porcine circovirus type 2*, PCV2), Cap-based subunit or VLP vaccines have attained significant success [16,17]. Furthermore, previous studies have predicted linear B-cell epitopes on the GoCV Cap protein, underscoring its potential as a vaccine candidate [11]. Prokaryotic expression systems offer a cost-effective and scalable approach for producing recombinant Cap antigens for vaccine development [18,19].

Building on these foundations, our research team first overcame technical barriers in GoCV isolation, successfully isolating the virus and establishing a gosling pathogenicity model, which has since served as a crucial technical platform for GoCV vaccine development and efficacy evaluation [20]. Since the presence of the N-terminal nuclear localization signal (NLS) of GoCV Cap protein impairs its expression efficiency in *Escherichia coli*, we constructed a prokaryotic expression system for the truncated GoCV Cap (GoCV-ΔCap) by deleting the NLS in this study to improve its expression efficiency in *E. coli* [21]. The purified protein was formulated with aluminum hydroxide as an adjuvant to prepare the vaccine, and its immunoprotective efficacy was systematically evaluated in the established gosling pathogenicity model, with evaluations encompassing clinical, pathological, virological, and immunological endpoints. Our team previously provided the first experimental evidence that GoCV infection significantly reduces the immune efficacy of NDV and AIV bivalent inactivated vaccines [20], with a specific emphasis on the vaccine’s capacity to alleviate GoCV-induced immunosuppression and its potential interference with routine vaccination regimens.

## 2. Materials and Methods

### 2.1. Preparation and Safety Evaluation of GoCV-ΔCap Protein Subunit Vaccine

To prepare the GoCV Cap subunit vaccine, a prokaryotic expression plasmid designated pET-30a-ΔCap was constructed (synthesized by GenScript Biotech Corp., Piscataway, NJ, USA) for subsequent purification and immunization studies. Derived from the pET-30a (+) vector, this recombinant plasmid was designed with a deletion of the N-terminal nuclear localization signal (NLS) to enhance expression efficiency in *E. coli*. The plasmid was transformed into *E. coli* BL21 (DE3) competent cells (TransGen Biotech Co., Ltd., Beijing, China), and transformed colonies were cultured in LB broth supplemented with 100 μg/mL kanamycin at 37 °C with shaking until the culture reached an OD600 of ~0.6–0.8. Protein expression was induced by adding 0.5 mM IPTG, followed by incubation at 37 °C for 5 h. Bacterial cells were harvested by centrifugation, resuspended in lysis buffer, and disrupted by sonication on ice. The lysate was clarified by centrifugation for subsequent protein purification. The GoCV-ΔCap protein was purified from SDS-PAGE gels via gel extraction [22], and the purified GoCV-ΔCap protein was probed with the Rabbit anti His-tag mAb (AE086, ABclonal Biotechnology Co., Ltd., Wuhan, China) to specifically recognize the target protein, confirming the identity of the purified product. Its concentration was determined using a BCA protein assay kit (Beyotime Biotech Co., Ltd., Shanghai, China). For immunization, the purified protein was emulsified with sterile aluminum hydroxide adjuvant (Inner Mongolia Xinhong Biotech Co., Ltd., Hohhot, China) at a 1:1 (*v*/*v*) ratio to formulate a 2% aluminum hydroxide-adjuvanted subunit vaccine, with final antigen concentrations of 75 µg/mL (high-dose) or 15 µg/mL (low-dose).

The study was approved by the Committee on Animal Ethics of Foshan University (Permission number: FOSU2021194501), and all experiments were conducted in accordance with the guidelines for the Care and Use of Laboratory Animals of the National Institutes of Health of the USA. Specifically, goslings were euthanized after anesthesia via the intraperitoneal injection of sodium pentobarbital at a dosage of 150 mg/kg for the animal experiments. In cases where goslings displayed symptoms, they were anesthetized with a low dose of sodium pentobarbital (50 mg/kg) before dissection was performed.

For safety evaluation, six 4-day-old healthy male White Goslings were randomly divided into two groups (*n* = 3 per group). The test group received a 375 µg/mL vaccine dose via intramuscular (i.m.) injection into the leg muscle, while the control group received an equal volume of phosphate-buffered saline (PBS; Beijing EallBio Biotech Co., Ltd., Beijing, China). Clinical signs (appetite, activity, general behavior) were monitored daily. All goslings were euthanized three weeks post-immunization for gross examination of internal organs and injection sites.

### 2.2. Vaccine Challenge Protection Trial

**Figure 1:**
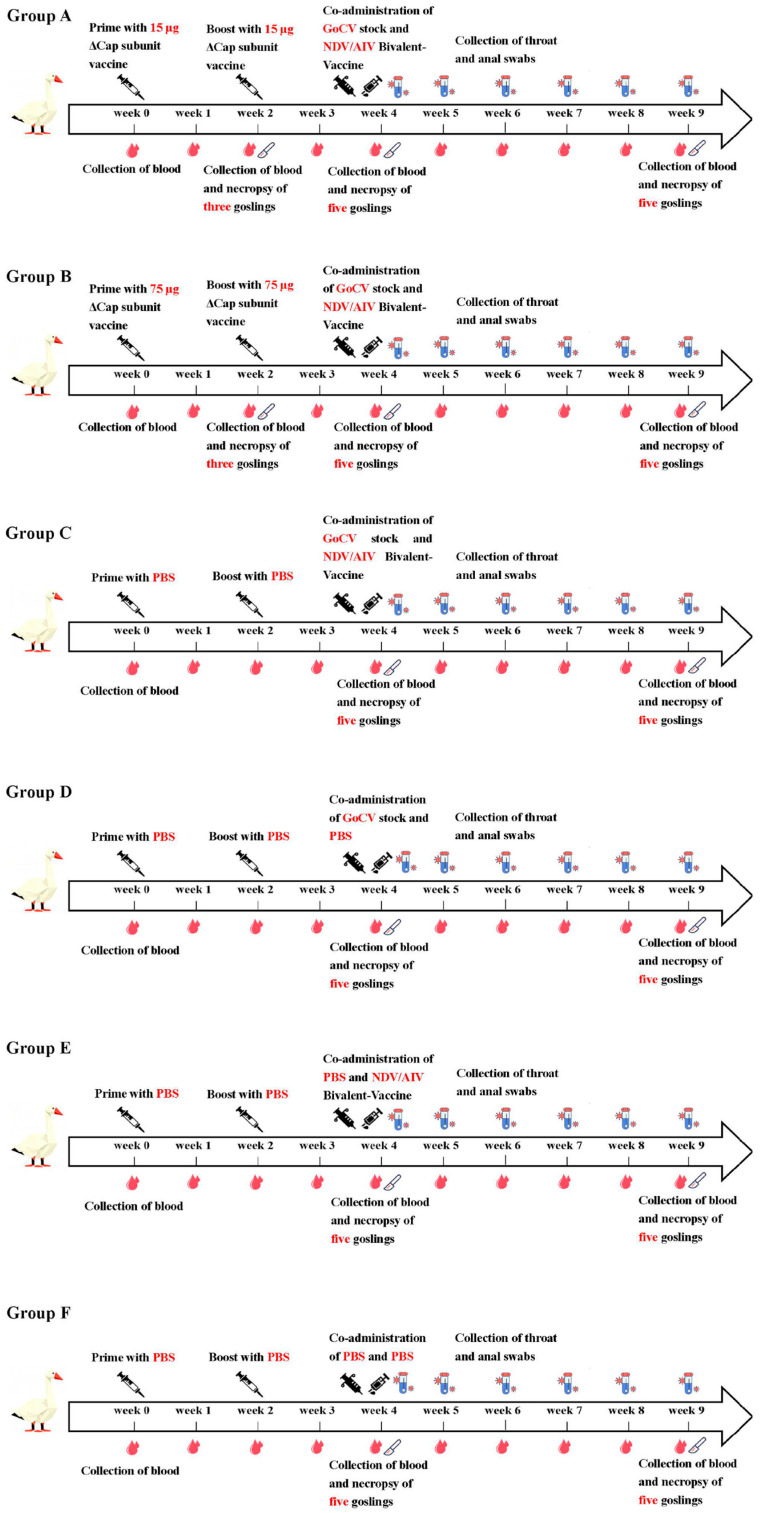
**Flow chart of immunoprotection test.** This schematic illustrates the timelines of primary and booster immunization with the GoCV-ΔCap subunit vaccine, GoCV challenge, and administration of the commercial NDV/AIV bivalent inactivated vaccine, as well as the corresponding time points for sample collection, for all experimental groups.

**Experimental Animal Grouping, Immunization, and Challenge Procedures.** Sixty-nine 4-day-old male White Goslings with no significant clinical signs and similar body weight were obtained from a commercial farm in Foshan, Guangdong. All goslings were confirmed GoCV-negative by PCR and ELISA, ensuring the absence of GoCV nucleic acid and serological antibodies. They were randomly assigned to six groups (10 goslings for Groups C, D, and E; 13 goslings for Groups A, B, and F; Table 1), with infected and control groups housed in separate rooms to prevent cross-contamination. All subsequent procedures followed randomized allocation principles. Groups A and B were immunized intramuscularly in the leg muscle with 1 mL of low-dose (15 µg) or high-dose (75 µg) GoCV-ΔCap vaccine, respectively, while Groups C-F received 1 mL of PBS instead. A boost immunization was administered 14 days post-primary immunization using the same formulation and dosage. For the challenge experiment, the GoCV strain GoCV/369/2020 (GenBank accession no. MT831925)-isolated and maintained in our laboratory, propagated to the third passage in primary goose embryo kidney (GEK) cells-was used, with a viral stock titer of 10^7^ genomic copies/mL as determined by qPCR. At 14 days post-boost immunization, goslings in Groups A-D were inoculated i.m. in the leg muscle with 1 mL of this viral stock, while Groups E and F received an equal volume of PBS. Concurrently, Groups A, B, C, and E received a subcutaneous neck injection of 1 mL of the commercial NDV/AIV bivalent inactivated vaccine, while Groups D and F received PBS. Group E served as the non-challenged vaccine control, and Group F as the absolute negative control. All surviving goslings were euthanized at 35 days post-challenge for sample collection. A schematic of the experimental design is shown in Figure 1. For clarity, time points related to GoCV-ΔCap priming and boosting are reported as days post-immunization (prime- or boost-dpi), those related to GoCV challenge as days post-challenge (dpc), and those related to NDV/AIV bivalent vaccination as days post-immunization of the NDV/AIV bivalent vaccine (bivac-dpi).

**Clinical and Pathological Examination.** Body weight and feather development of goslings were monitored daily from 0 to 35 dpc. At 35 dpc, five goslings per group were randomly euthanized, and lymphoid organs (bursa of Fabricius, thymus, spleen, bone marrow) were aseptically collected for gross and histopathological evaluation. Tissue samples were fixed in 10% neutral-buffered formalin and sent to a commercial service provider (Guangzhou Maike Biotech Co., Ltd., Guangzhou, China) for paraffin embedding, sectioning, and hematoxylin-eosin (HE) staining. Additionally, lymphoid organ samples collected at 35 dpc were stored at −80 °C for subsequent nucleic acid extraction.

**Viral Load Quantification.** Tissue samples were collected at two time points: pre-challenge and 35 dpc. At each time point, five goslings per group were randomly selected and euthanized, and lymphoid organs (bursa of Fabricius, thymus, spleen, bone marrow) were aseptically collected. Throat and cloacal swabs were collected from all goslings at 0, 7, 14, 21, 28, and 35 dpc. Viral DNA was extracted from tissue homogenates and swab samples using a commercial DNA extraction kit (Guangzhou Magen Biotech Co., Ltd., Guangzhou, China). Absolute quantification of GoCV genomic copies was performed via SYBR-based qPCR targeting the Rep gene on a QuantStudioTM 5 Real-Time PCR system (Thermo Fisher Scientific, Waltham, MA, USA), using forward primer 5′-GGTCTGCCGATAACTGA-3′ and reverse primer 5′-GGCCGACCAATCAGAACGA-3′. Viral genome copy numbers were calculated using an instrument-specific standard curve (y = −3.1786x + 36.434, R^2^ = 0.9954).

**Cytokine Expression Analysis.** At 14 prime-dpi and 14 boost-dpi, three randomly selected goslings from Groups A and B were euthanized, and lymphoid organ samples (bursa of Fabricius, thymus, spleen, bone marrow) were collected. Total RNA was extracted using a commercial RNA extraction kit (Chengdu Forgene Biotech Co., Ltd., Chengdu, China) and reverse-transcribed into complementary DNA (cDNA) with a cDNA synthesis kit (Suzhou Kangrun Jingxing Biotech Co., Ltd., Suzhou, China). mRNA expression levels of IFN-γ, IL-2, IL-4, and IL-6 were quantified via SYBR-based qRT-PCR using GAPDH as the internal reference gene. Relative expression levels were computed using the 2-∆∆Ct method, and dissociation curve analysis was performed to verify amplification specificity. Each reaction was run in triplicate. Primer sequences are listed in Table 2.

**Serological Assays.** GoCV-specific antibodies in gosling sera were detected using a previously established indirect ELISA [22]. Serum samples were collected from Groups A, B, and F at 0, 7, 14, 21, and 28 days post-primary immunization (dpi). At 28 dpi, goslings were challenged with GoCV, and this time point was simultaneously defined as 0 dpc. Subsequently, serum samples from all groups were collected at 0, 7, 14, 21, 28, and 35 dpc. Briefly, ELISA plates were coated with recombinant GoCV-ΔCap protein at 4 μg/mL and blocked with 2% skim milk (BBI Life Sciences Co., Ltd., Shanghai, China). Gosling sera diluted 1:100 were used as the primary antibody, followed by horseradish peroxidase (HRP)-conjugated goat anti-duck IgG (H+L) (5220-0296, SeraCare Life Sciences, Inc. (KPL), Milford, MA, USA) diluted 1:1000 as the secondary antibody. Each step was followed by six washes with PBST. Color development and termination were performed using TMB single-component substrate and ELISA stop solution (Beijing Solarbio Science & Technology Co., Ltd., Beijing, China), respectively. Optical density values were measured at 450 nm using a microplate reader, with a cutoff value of OD450 = 0.147 for the iELISA.

For assessing antibody responses to the NDV/AIV bivalent vaccine, serum samples were collected at 0, 7, 14, 21, 28, and 35 bivac-dpi. Hemagglutination inhibition (HI) assays were performed per standard procedures: serial twofold-diluted serum samples were incubated with 4 hemagglutination units (HAU) of the respective viral antigen, followed by the addition of a 1% chicken red blood cell suspension. HI titers were recorded as the highest serum dilution that completely inhibited hemagglutination, and geometric mean titers (GMTs) were calculated for each group.

### 2.3. Statistical Analysis

Each gosling served as the experimental unit. Statistical analyses were performed using the General Linear Model (GLM) procedure in SPSS software (version 25.0). The statistical model included the fixed effect of treatment group; for repeated measurements (e.g., body weight, antibody titers), the model also included fixed effects of time and treatment × time interaction. Differences were considered statistically significant at *p* < 0.05. Results are presented as mean ± SEM.

## 3. Results

### 3.1. Protein Expression and Safety Performance of GoCV-ΔCap Vaccine

**Figure 2:**
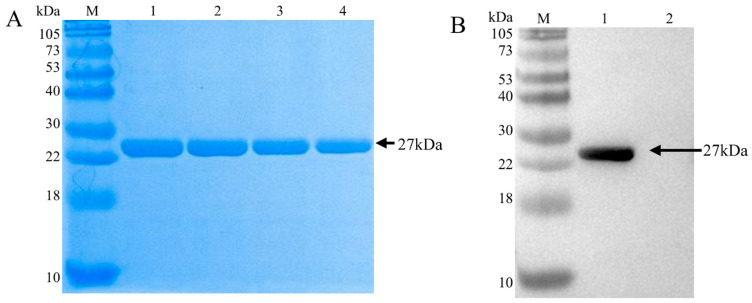
**Purification and specific identification of recombinant GoCV-ΔCap protein.** (**A**) SDS-PAGE analysis of GoCV-ΔCap protein purified by gel extraction. M: Protein molecular weight marker; Lanes 1–4: Purified GoCV-ΔCap protein obtained by gel extraction. (**B**) Western blot identification of GoCV-ΔCap protein using a His-tag antibody. M: Protein molecular weight marker; Lane 1: Purified GoCV-ΔCap protein; Lane 2: Negative control (*E. coli* BL21 (DE3) lysate without recombinant plasmid). The band indicated by the arrow is the expected protein.

The GoCV-ΔCap protein was expressed and purified in accordance with the method described in Section 2.1. The purification efficiency was then verified by SDS-PAGE (Figure 2A), and the results showed a high-purity protein band at 27 kDa. Furthermore, Western blot analysis was performed on this protein sample, and the target protein was specifically recognized using a His-tag antibody (Figure 2B), confirming the correct expression of GoCV-ΔCap.

The GoCV-ΔCap subunit vaccine was prepared according to the method described in Section 2.1. Goslings inoculated with a dose of 375 µg/mL of this vaccine exhibited no adverse clinical signs, behavioral abnormalities, or mortality throughout the entire observation period. Necropsy showed no gross pathological lesions in the visceral organs or at the injection sites.

### 3.2. Dose-Dependent Protection of GoCV Subunit Vaccine Against Growth Suppression

**Figure 3:**
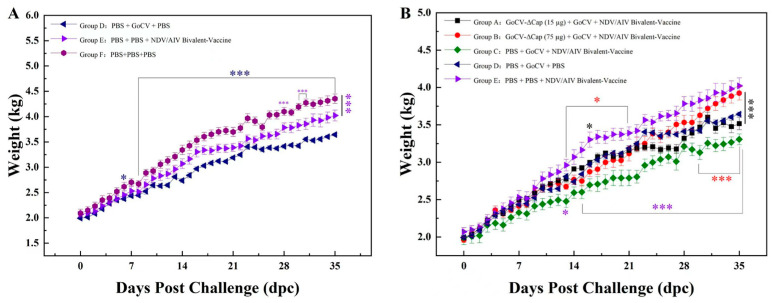
**Body weight dynamics of goslings in each experimental group following GoCV challenge.** Goslings were immunized at 4 and 18 days of age, with concurrent GoCV challenge and NDV/AIV bivalent vaccine administration at 14 boost-dpi. Body weight was monitored periodically post-challenge. (**A**) Comparison of the GoCV-infected control group (Group D) and the bivalent-vaccinated control group (Group E) with the blank negative control group (Group F); significant differences between Groups D and F are denoted by blue asterisks, and significant differences between Groups E and F are denoted by purple asterisks. (**B**) Comparative analysis of the vaccinated groups (Groups A and B), the control groups (Groups C and E), and the GoCV-infected control group (Group D); significant differences involving Group A (black curve) are denoted by black asterisks, those involving Group B (red curve) are denoted by red asterisks, and those involving Group E (purple curve) are denoted by purple asterisks. Data are expressed as mean ± SEM. Asterisks denote statistically significant differences (* *p* < 0.05; *** *p* < 0.001).

GoCV challenge markedly inhibited body weight gain in goslings. Challenged goslings in Group D showed progressive growth retardation, with a significant difference compared with the negative control Group F starting at 6 dpc; the difference reached an extremely significant level from 8 to 35 dpc (*p* < 0.001). The body weight of the non-challenged control Group E, which only received the NDV/AIV bivalent vaccine, was slightly lower than that of Group F, with significant differences observed only at 28, 30, 31, and 35 dpc (*p* < 0.001). This mild growth retardation in Group E may result from moderate metabolic consumption and immune response expenditure induced by routine single administration of the bivalent vaccine. In the absence of viral challenge, such processes may impose a slight physiological burden on the normal growth of the host (Figure 3A).

Furthermore, infected goslings administered only the NDV/AIV bivalent vaccine (Group C) showed greater weight loss than the non-challenged vaccine control (Group E), with differences observed from 13 dpc (*p* < 0.05) and marked differences from 15 to 35 dpc (*p* < 0.001). Vaccination with the GoCV-ΔCap subunit vaccine mitigated this growth suppression in a dose-dependent manner. At 35 dpc, the body weight of the high-dose group (Group B, 75 µg/mL) was significantly higher than that of the low-dose group (Group A, 15 µg/mL) (*p* < 0.001). Notably, from 30 to 35 dpc, Group B exceeded the weight of the infected control (Group C, *p* < 0.001), whereas Group A showed no significant improvement over Group C. Both vaccinated groups remained lighter than the healthy vaccine control (Group E) during the mid-infection period (13–21 dpc for Group B, *p* < 0.05; 16–35 dpc for Group A, *p* < 0.05). Importantly, Group B showed a significant difference from Group E only between 13 and 21 dpc, after which its body weight gradually approached that of Group E (Figure 3B).

### 3.3. Superior Protection of High-Dose Vaccine Against GoCV-Induced Organ Pathology

**Figure 4:**
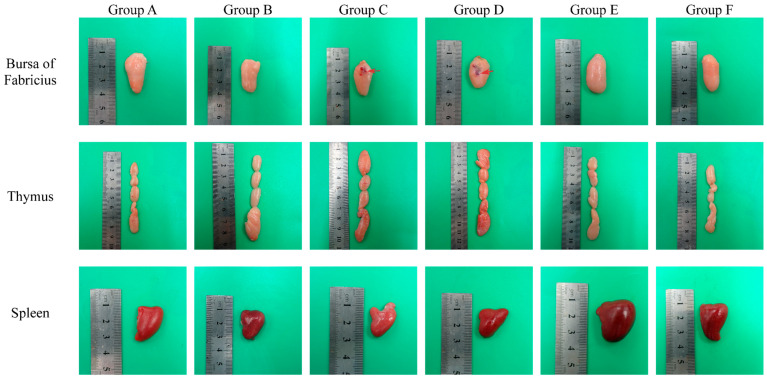
**Gross pathological changes in lymphoid organs across groups at 35 days post-challenge (dpc).** Representative gross pathology images from each group demonstrate the spectrum of lesion severity. Red arrows denote sites of bursal hemorrhage.

Gross pathological examination of lymphoid organs at 35 dpc revealed that GoCV infection, either in the infected control group (Group D) or the NDV/AIV bivalent vaccine-infected group (Group C), induced severe lesions, including bursal atrophy with hemorrhage (red arrow), thymic hypertrophy accompanied by hemorrhage, and splenic atrophy. In contrast, tissues from the negative control (Group F) and vaccine-only control (Group E) appeared normal, confirming that the NDV/AIV bivalent vaccine alone did not cause pathological changes (Figure 4). Vaccination with the GoCV-ΔCap subunit vaccine significantly mitigated these gross pathological lesions, with a clear dose-dependent protective effect. Protective efficacy was most pronounced in the bursa of Fabricius: the incidence of localized atrophy was completely eliminated in the high-dose group (Group B: 0/5), compared to 60% in the low-dose group (Group A: 3/5). Similarly, thymic lesions (partial lobular enlargement and hemorrhage) were reduced by half, from 80% (4/5) in Group A to 40% (2/5) in Group B. A reduction in splenic atrophy incidence was also evident in the high-dose group (Group B: 1/5) compared to the low-dose group (Group A: 2/5). Collectively, these data demonstrate that the high-dose vaccine conferred superior protection against GoCV-induced gross lesions across primary lymphoid organs (Table 3).

**Figure 5:**
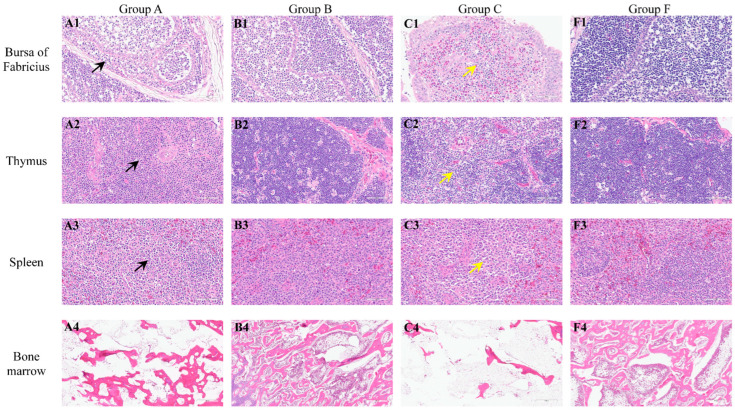
**Histopathological alterations in lymphoid tissues of each group at 35 days post-challenge (dpc).** Hematoxylin and eosin (HE) staining was conducted at 400× magnification for the bursa of Fabricius, thymus, and spleen, and at 40× magnification for bone marrow. (**A1**–**A4**) Group A, showing moderate lymphoid tissue damage. Black arrows in (**A1**–**A3**) indicate mild reduction in bursal lymphocytes with interstitial edema, slight loss of thymic cortical lymphocytes, and enlarged, disorganized splenic corpuscles, respectively. (**B1**–**B4**) Group B, showing well-preserved lymphoid tissue architecture, closely resembling normal morphology, with intact bursal follicles, orderly thymic cortical lymphocytes, normal splenic corpuscles, and preserved bone marrow cellularity. (**C1**–**C4**) Group C, demonstrating severe pathological alterations. Yellow arrows in (**C1**–**C3**) indicate bursal follicular hemorrhage, atrophy, necrosis, and heterophilic infiltration; depletion of thymic cortical lymphocytes; and enlarged splenic corpuscles, respectively. (**F1**–**F4**) Group F, serving as the negative control, displayed normal lymphoid tissue architecture, characterized by intact bursal follicles, orderly cellular arrangement, and the absence of cellular degeneration, interstitial edema, vascular dilation, or inflammatory infiltration.

Consistent with gross pathological findings, histopathological analysis confirmed dose-dependent preservation of lymphoid tissue integrity (representative histology images of the severe lesion Group D and normal Group E are shown in Supplementary Material Figure S1). Group C exhibited severe pathological alterations, characterized by bursal follicular hemorrhage, atrophy, necrosis, and heterophilic infiltration; thymic cortical lymphocyte depletion; enlarged splenic corpuscles (yellow arrow); and hypocellular bone marrow with adipose replacement (Figure 5C1–C4). In contrast, vaccination markedly improved histological outcomes. Compared to the normal morphology of the negative control group (Group F; Figure 5F1–F4), Group A exhibited moderate lymphoid damage, including mild bursal lymphocyte reduction with interstitial edema, slight thymic cortical lymphocyte loss, enlarged and disorganized splenic corpuscles (black arrow), and reduced bone marrow cellularity with mild adipocyte infiltration (Figure 5A1–A4). Group B, however, displayed well-preserved lymphoid architecture across all examined tissues (Figure 5B1–B4), closely resembling the normal morphology of control groups.

### 3.4. Vaccination Reduces Tissue Viral Burden and Detection Rate

**Figure 6:**
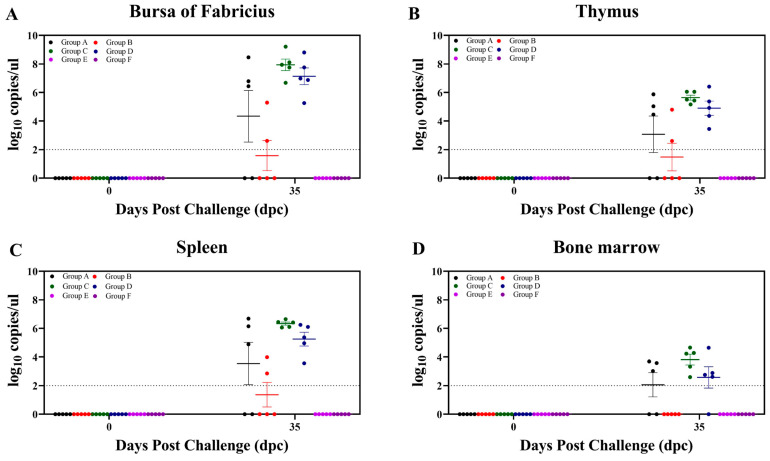
**Viral load comparisons in lymphoid tissues of goslings pre-challenge and at 35 days post-challenge (dpc).** (**A**–**D**) Viral loads in the bursa of Fabricius, thymus, spleen, and bone marrow, respectively. The dashed line denotes the lower limit of detection for GoCV qPCR. Data are expressed as mean ± SEM.

At 35 dpc, viral loads in the bursa of Fabricius, thymus, spleen, and bone marrow were quantified by qPCR. All tissue samples tested negative for GoCV prior to viral challenge. Following GoCV challenge, high viral loads were detected in all infected control goslings (Groups C and D; 5/5), with the highest copy numbers in the bursa of Fabricius and the lowest in the bone marrow (except for one Group D sample below the detection limit; Figure 6).

Vaccination induced a clear, dose-dependent reduction in both the prevalence and magnitude of viral tissue burden. GoCV DNA was detected in 3/5 goslings in the low-dose group (Group A) and 2/5 goslings in the high-dose group (Group B). Notably, no positive samples were detected in the bone marrow of Group B goslings. Furthermore, viral loads in all four lymphoid tissues were consistently and significantly lower in Group B compared to Group A (Figure 6). These findings indicate that the high-dose vaccine regimen effectively controlled GoCV replication and limited viral dissemination within primary lymphoid organs.

### 3.5. Vaccination Alters Viral Shedding Dynamics and Reduces Viral Load

**Figure 7:**
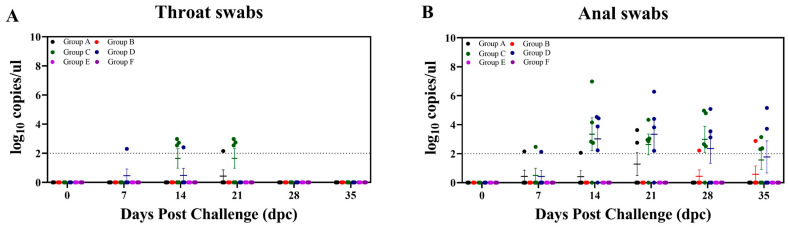
**Viral shedding dynamics post-GoCV challenge.** (**A**) Viral loads in throat swabs. (**B**) Viral loads in cloacal swabs. The dashed line denotes the lower limit of detection for GoCV qPCR. Data are expressed as mean ± SEM.

Viral shedding was monitored weekly via throat and cloacal swabs. No viral shedding was detected in uninfected control goslings (Groups E and F). In contrast, infected control goslings (Groups C and D) exhibited sustained viral excretion: cloacal shedding persisted until 35 dpc, while throat shedding lasted until 21 dpc, peaking between 14 and 21 dpc (Figure 7).

Vaccination markedly reduced viral shedding in a dose-dependent manner. In the low-dose group (Group A), cloacal swabs tested positive from 7 to 21 dpc, and one throat swab tested positive at 21 dpc. In contrast, the high-dose group (Group B) showed delayed onset of viral shedding, with only one cloacal swab testing positive between 28 and 35 dpc and no detectable throat shedding throughout the observation period. Additionally, viral loads in positive samples from Group B were substantially lower than those in Group A (Figure 7). These results demonstrate that the high-dose vaccination regimen markedly reduced viral shedding frequency, viral load, throat shedding, and overall shedding duration. Only low-level cloacal viral detection was observed in a single gosling at later time points.

### 3.6. Vaccination Induces Robust and Sustained GoCV-Specific Antibody Response

**Figure 8:**
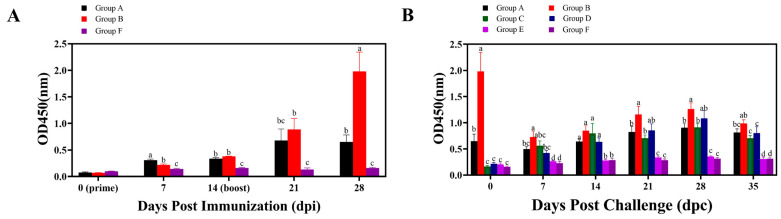
**Detection of GoCV-ΔCap-specific antibodies.** (**A**) Antibody levels in goslings over 0–28 days post-primary immunization (dpi); (**B**) Antibody levels in goslings over 0–35 days post-challenge (dpc). Data are expressed as mean ± SEM. Significant differences among experimental groups at the same time point are denoted by letters “a” to “d”: groups sharing the same letter show no significant differences, while those with different letters indicate statistically significant differences.

Serum levels of GoCV-ΔCap-specific IgG were measured by indirect ELISA. No GoCV-specific antibodies were detected in uninfected control goslings (Group F). In vaccinated groups (A and B), GoCV-ΔCap-specific IgG became detectable as early as 7 dpi and increased steadily thereafter. A marked anamnestic response was observed following the boost immunization at 14 dpi. From this point onward, goslings in the high-dose group (Group B) maintained consistently higher antibody titers than those in the low-dose group (Group A). At 14–28 dpi, antibody levels in both vaccinated groups were significantly higher than those in the uninfected control (Group F), and at 28 dpi, the antibody titer of Group B was significantly higher than that of Group A (*p* < 0.05). Quantitatively, at 14 and 28 dpi, antibody levels in Group A were 2.11- and 4.11-fold higher than those in Group F, respectively. In contrast, levels in Group B were 2.37- and 12.52-fold higher than those in Group F, and 3.05-fold higher than those in Group A at 28 dpi (Figure 8A).

Following GoCV challenge at 28 dpi, antibody titer dynamics changed markedly. Vaccinated goslings (Groups A and B) exhibited a transient decline in antibody titers at 7 dpc, followed by a rebound reflecting effective reactivation of immune memory. In contrast, infected control goslings (Groups C and D) displayed a typical primary antibody response, with titers rising from 7 dpc and peaking at 28 dpc. From 21 to 35 dpc, antibody titers in the high-dose group (Group B) remained significantly higher than those in the infected control group (Group C) (*p* < 0.05), indicating a more sustained humoral response. By the end of the trial (35 dpc), Group B maintained the highest antibody levels among all challenged groups, further confirming the robust and long-lasting immunity conferred by vaccination (Figure 8B).

### 3.7. Vaccine-Induced Cytokine Responses

To evaluate the cellular immune response induced by the GoCV-ΔCap subunit vaccine, we detected the mRNA expression levels of IFN-γ, IL-2, IL-4 and IL-6 in lymphoid organs at 14 days post-primary immunization (14 prime-dpi) and 14 days post-booster immunization (14 boost-dpi, pre-challenge).

The results showed that compared with the PBS control group (Group C) at the same time points, both the high-dose (Group B) and low-dose (Group A) GoCV-ΔCap vaccine groups significantly upregulated the expression of Th1-type cytokines (IFN-γ, IL-2) and Th2-type cytokines (IL-4, IL-6), suggesting that the vaccine induces a cellular immune response. However, no statistically significant difference in cytokine expression levels was observed between the high-dose and low-dose groups at either time point. Detailed bar graphs and statistical analyses are provided in Supplementary Figure S2.

### 3.8. Overcoming GoCV-Induced NDV/AIV Vaccine Interference with the Subunit Vaccine

To assess whether GoCV infection impairs the immunogenicity of routine vaccinations, HI antibody responses to the co-administered AIV and NDV bivalent vaccine were evaluated. As expected, goslings in control groups not receiving the bivalent vaccine (Groups D and F) showed no detectable HI antibodies.

**Figure 9:**
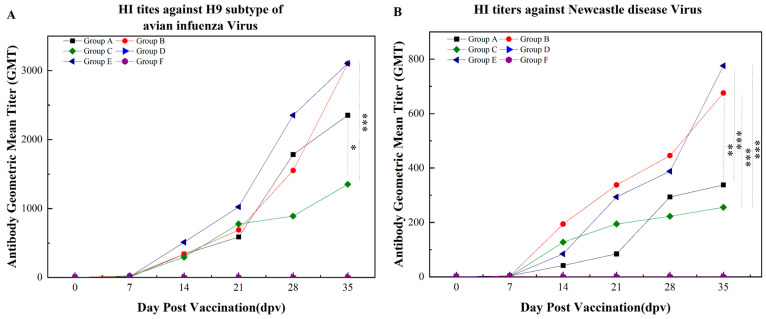
**Hemagglutination inhibition (HI) antibody responses to the NDV/AIV bivalent vaccine across experimental gosling groups.** Geometric mean titers (GMTs) against (**A**) Avian influenza virus (AIV) and (**B**) Newcastle disease virus (NDV) are presented. Data are expressed as mean ± SEM. Asterisks denote statistically significant differences (* *p* < 0.05, ** *p* < 0.01, *** *p* < 0.001).

At 35 dpc, for the AIV component, goslings infected with GoCV at the time of bivalent vaccination (Group C) developed significantly lower HI antibody titers than the non-infected, bivalent-vaccinated control group (Group E) (*p* < 0.001). Vaccination with the GoCV-ΔCap subunit vaccine markedly mitigated this interference: the high-dose group (Group B) achieved AIV HI antibody levels comparable to Group E, while the low-dose group (Group A) exhibited significantly higher HI antibody titers than Group C (*p* < 0.05) but remained lower than Groups B and E (Figure 9A). For the NDV component, a more pronounced immunosuppressive effect of GoCV infection was observed: HI antibody GMTs in Group C were significantly lower than those in Group E (*p* < 0.001). The GoCV-ΔCap vaccine again demonstrated a clear dose-dependent protective effect: the high-dose group (Group B) achieved NDV HI antibody levels comparable to Group E and significantly higher than those in Group C (*p* < 0.001). The low-dose group (Group A) also showed significant improvement compared to Group C, though its antibody titers remained lower than those in Groups B (*p* < 0.01) and E (*p* < 0.001) (Figure 9B).

In summary, these results confirm that GoCV infection suppresses humoral immune responses to heterologous vaccines, while immunization with the GoCV-ΔCap subunit vaccine effectively alleviates this immunosuppression in a dose-dependent manner, thereby preserving the immunogenicity of concurrently administered vaccines.

## 4. Discussion

The lack of a commercially available vaccine against *goose circovirus* (GoCV) has long been a major barrier to controlling the economic losses caused by goose circovirus disease (GoCVD), a predicament exacerbated by two-decade-long technical bottlenecks: low efficiency of in vitro viral isolation and culture, and the absence of a reliable animal pathogenic model. These limitations have severely hindered GoCV pathogenesis research and vaccine development. In a critical breakthrough, our research team successfully overcame both challenges by establishing a stable primary goose embryo kidney (GEK) cell culture system for efficient GoCV propagation and a standardized gosling pathogenic model, thereby providing a robust technical platform for GoCV vaccine evaluation [20]. Building on this innovative framework, the present study represented the first attempt to develop a GoCV subunit vaccine and systematically assess its immunoprotective efficacy, which is specifically a prokaryotically expressed vaccine based on a truncated Capsid protein (GoCV-ΔCap).

Our results demonstrate that the high-dose (75 µg) vaccine formulation provided substantial protection against GoCV challenge by reducing viral replication, mitigating pathological damage, and importantly alleviating virus-induced immunosuppression. The protective efficacy observed aligns with the established principle that the Capsid protein serves as the principal protective antigen for *circoviruses*, as demonstrated in porcine *circovirus* type 2 (PCV2) vaccine studies [23,24]. While sterilizing immunity was not achieved, the high-dose vaccine significantly reduced viral loads in lymphoid tissues and diminished both the duration and magnitude of viral shedding. The incomplete immune protection may partly reflect interference from residual maternal antibodies, as goslings were immunized at 4 days of age, when maternal antibodies remain relatively high. Maternal antibodies in geese typically wane between 2 and 4 weeks of age, and early vaccination during this period may transiently inhibit the recognition of vaccine antigens and delay the development of active immunity [25,26]. As these antibodies declined, however, vaccine-induced immune responses became more pronounced, consistent with the gradual increase in antibody titers observed after the booster. This level of control, shifting infection toward a subclinical state, is consistent with the protective goals of many successful veterinary vaccines [27,28]. The reduction in both the prevalence and severity of lymphoid lesions in vaccinated goslings further confirms the biological relevance of this viral containment.

A robust and balanced immune response is critical for effective antiviral protection [29,30]. The GoCV-ΔCap vaccine induced high titers of capsid-specific protective antibodies and promoted a mixed Th1/Th2 cytokine profile, indicative of coordinated humoral and cellular immune responses. Notably, the transient decline in antibody titers observed post-challenge warrants consideration. Similar phenomena have been reported in other viral systems and may reflect transient consumption of circulating antibodies through binding to viral antigens and immune complex formation, as well as redistribution of immune cells to sites of infection [31,32]. This dynamic response likely reflected active engagement of the adaptive immune system rather than its failure.

A key finding of this study is the vaccine’s ability to mitigate GoCV-induced immunosuppression. The impaired antibody responses to the NDV/AIV bivalent vaccine in GoCV-infected controls (Group C) clearly illustrate the immunosuppressive nature of GoCV. In contrast, the high-dose GoCV-ΔCap vaccine group (Group B) mounted HI antibody responses comparable to those of non-infected, vaccinated controls (Group E). These findings provide direct experimental evidence that controlling GoCV replication via vaccination preserves immunocompetence against concurrent pathogens, a finding with important implications for field settings where polymicrobial infections are prevalent.

It is noteworthy that feathering disorders, a previously reported clinical sign of GoCVD, were not observed in this study. This discrepancy with prior pathogenicity studies is likely attributable to the later challenge time (32 days of age) in our immunization model, which occurred after the most vulnerable period of feather follicle development. Thus, feather development was not considered a reliable clinical endpoint under the present experimental conditions.

In conclusion, this study provides comprehensive evidence that the prokaryotically expressed GoCV-ΔCap subunit vaccine, particularly at the 75 µg dose, represents a promising candidate for controlling GoCVD. Its development, rooted in our breakthroughs in GoCV isolation, culture, and pathogenic modeling, not only addresses the major pathological and economic challenges posed by GoCV but also offers critical technical support and valuable references for subsequent GoCV vaccine research. Future work should seek to validate field efficacy, define immune correlates of protection, assess cross-protective activity against heterologous strains, optimize immunization schedules, and evaluate industrial-scale feasibility and adjuvant formulations.

## Figures and Tables

**Table 1:** Vaccination Doses and Protocols.

Group No.	Inoculum	Number of Animals
Group A	GoCV-ΔCap (15 µg) + GoCV + NDV/AIV Bivalent-Vaccine	13
Group B	GoCV-ΔCap (75 µg) + GoCV + NDV/AIV Bivalent-Vaccine	13
Group C	PBS + GoCV + NDV/AIV Bivalent-Vaccine	10
Group D	PBS + GoCV + PBS	10
Group E	PBS + PBS + NDV/AIV Bivalent-Vaccine	10
Group F	PBS + PBS + PBS	13

**Notes:** GoCV-ΔCap: subunit vaccine formulated with the truncated capsid (Cap) protein of goose circovirus. NDV/AIV bivalent vaccine: commercial inactivated vaccine against Newcastle disease virus and H9-subtype Avian influenza virus.

**Table 2:** Primers for quantitative PCR analysis.

Cytokine	Forward Primer (F)	Reverse Primer (R)	bp
IFN-γ	GCCACACATCAAAAACCTGTCT	GGAGACTGGCTCCTTTTCCTT	207
IL-2	ACCGAGAGCTGACCAACTTT	ATCACCCACACTAAGAGCAT	177
IL-4	GGCATCTACCTCAACTTGCT	CTCTTTCGCTACTCGTTGGA	NA
IL-6	AGATGGTGATAAATCCTGATGA	CGGTTTTCTCCATAAATGAAGT	150
GAPDH	CATCTTCCAGGAGCGCGACC	AGACACCGGTGGACTCCACA	80

**Table 3:** Gross pathological alterations observed in goslings following GoCV challenge.

Organ	Gross Pathological Manifestations	Lesion Ratio	
		Group A	Group B
Bursa of Fabricius	Localized atrophy	3/5	0/5
Thymus	Partial lobular enlargement and hemorrhage	4/5	2/5
Spleen	Localized atrophy	2/5	1/5

## Data Availability

The GoCV/369/2020 strain used in this study is openly available in the GenBank database under the accession number: MT831925.

## Supplementary Materials

The following supporting information can be downloaded at: https://www.mdpi.com/article/10.3390/microorganisms14061227/s1, Figure S1: Representative histopathological images of severely lesioned Group D and normal Group E at 35 days post-challenge (dpc); Figure S2: Cytokine Expression in Lymphoid Tissues Induced by GoCV-ΔCap Subunit Vaccine.

## Abbreviations

The following abbreviations are used in this manuscript:

GoCV	Goose circovirus
Cap	Capsid protein
ΔCap	Truncated capsid protein
NLS	Nuclear localization signal
NDV	Newcastle disease virus
AIV	Avian influenza virus
HI	Hemagglutination inhibition
GMT	Geometric mean titer
GEK	Goose embryo kidney
