# Supplementary material for: Immunoevaluation of a Prokaryotic-Expressed Goose Circovirus Capsid Subunit Vaccine
Source: Microorganisms. 2026 May 29;14(6):1227. doi: 10.3390/microorganisms14061227 (PMC13304447; doi:10.3390/microorganisms14061227)
Supplement: Supplementary file 1 [file microorganisms-14-01227-s001.zip › Figure S1.pdf]

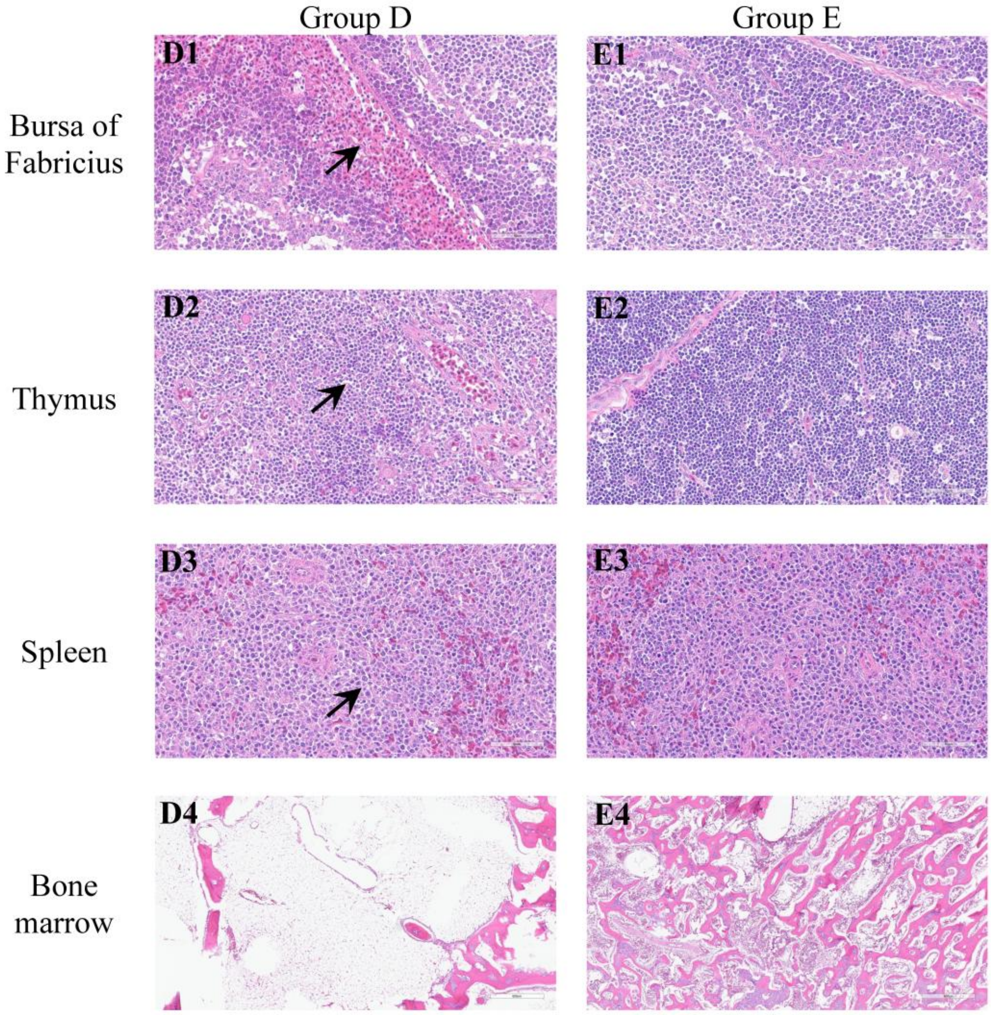

Figure S1. Representative histopathological images of severely lesioned Group D and normal Group E at 35 days post-challenge (dpc). Hematoxylin and eosin (HE) staining was conducted at 400× magnification for the bursa of Fabricius, thymus, and spleen, and at 40× magnification for bone marrow. (D1–D4) Group D, demonstrating severe pathological alterations. Black arrows in D1–D3 indicate bursal follicular hemorrhage, atrophy, necrosis, and heterophilic infiltration; depletion of thymic cortical lymphocytes; and enlarged splenic corpuscles, respectively. (E1–E4) Group E, serving as the negative control, displayed normal lymphoid tissue architecture, characterized by intact bursal follicles, orderly cellular arrangement, and the absence of cellular degeneration, interstitial edema, vascular dilation, or inflammatory infiltration.
