# Supplementary material for: Immunoevaluation of a Prokaryotic-Expressed Goose Circovirus Capsid Subunit Vaccine
Source: Microorganisms. 2026 May 29;14(6):1227. doi: 10.3390/microorganisms14061227 (PMC13304447; doi:10.3390/microorganisms14061227)
Supplement: Supplementary file 1 [file microorganisms-14-01227-s001.zip › Figure S2.pdf]

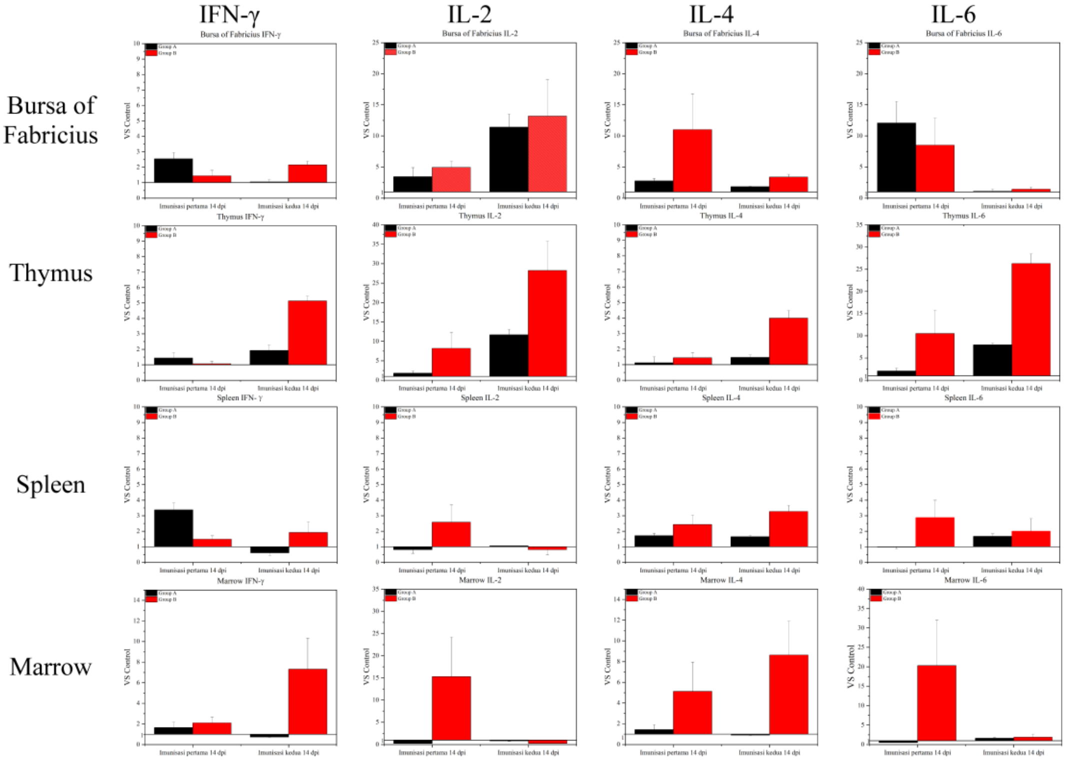

Figure S2. Cytokine Expression in Lymphoid Tissues Induced by GoCV-ΔCap Subunit Vaccine. The mRNA expression levels of IFN-γ, IL-2, IL-4 and IL-6 in various lymphoid tissues of goslings were detected at 14 days post-primary immunization (14 prime-dpi) and 14 days post-booster immunization (14 boost-dpi), and compared with those in the PBS negative control group (Group F). Data are expressed as mean ± standard error of the mean (mean ± SEM). The Y-axis represents the fold change of target gene expression relative to the control group.
