# Supplementary figures and images for: Immunoevaluation of a Prokaryotic-Expressed Goose Circovirus Capsid Subunit Vaccine
Source: Microorganisms. 2026 May 29;14(6):1227. doi: 10.3390/microorganisms14061227 (PMC13304447; doi:10.3390/microorganisms14061227)

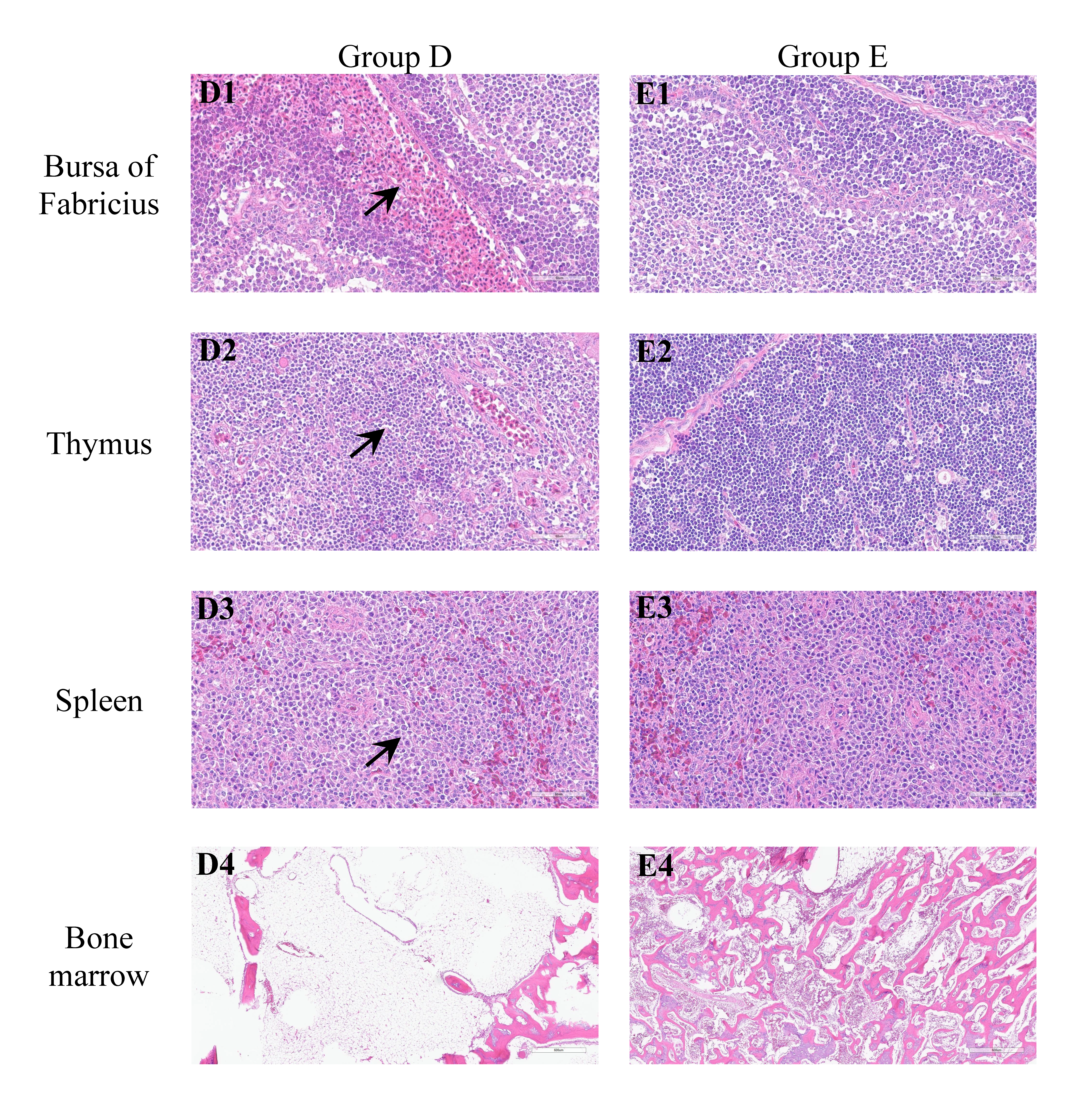

Supplement: Supplementary file 1 [file microorganisms-14-01227-s001.zip › Figure S1.png]

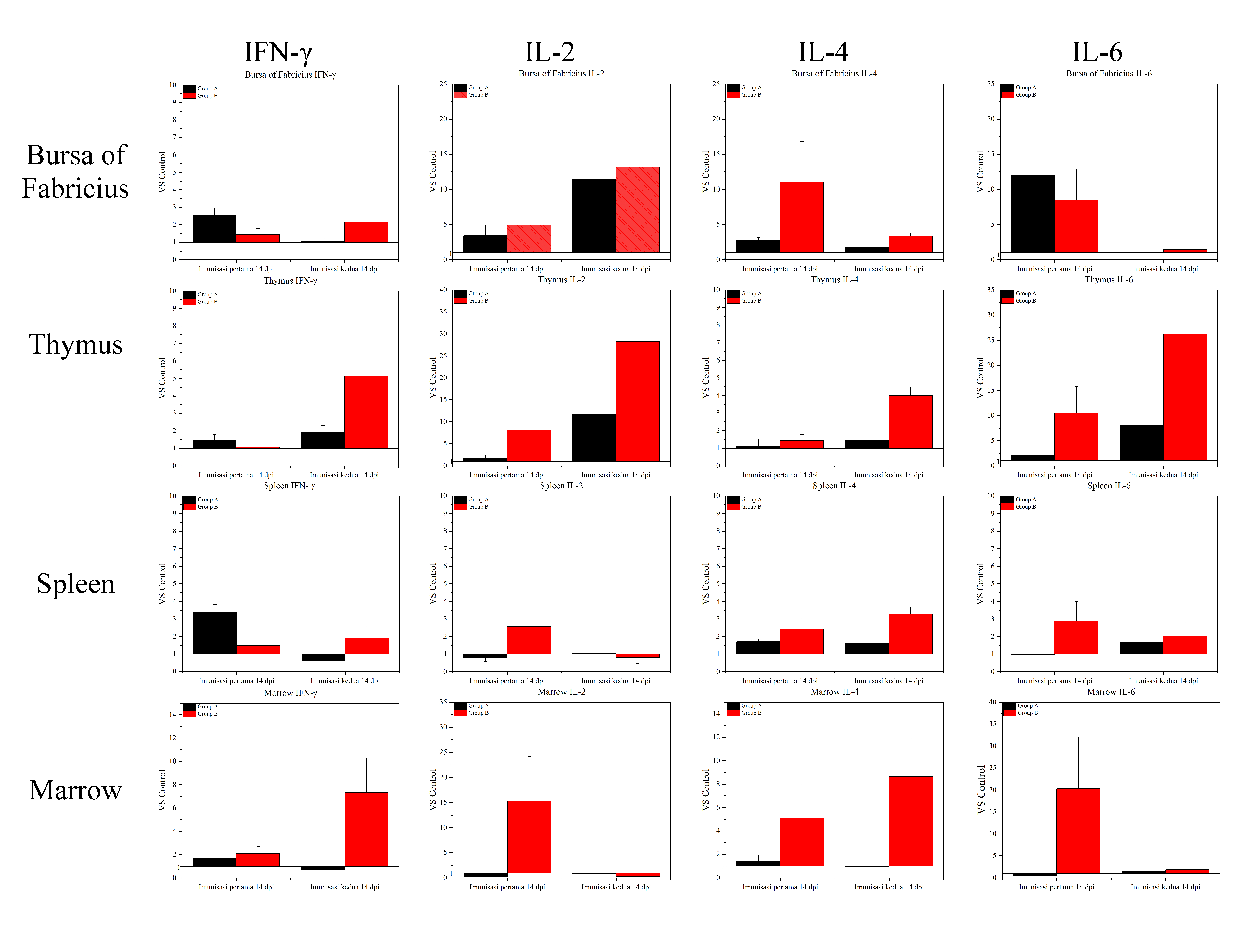

Supplement: Supplementary file 1 [file microorganisms-14-01227-s001.zip › Figure S2.png]
